# How to make DNA methylome wide association studies more powerful

**DOI:** 10.2217/epi-2016-0017

**Published:** 2016-04-07

**Authors:** Xinyi Lin, Sheila Barton, Joanna D Holbrook

**Affiliations:** 1 Singapore Institute for Clinical Sciences (SICS), Agency for Science & Technology Research (A*STAR), Brenner Centre for Molecular Medicine, 30 Medical Drive, 117609, Singapore; 2 MRC Lifecourse Epidemiology Unit, Faculty of Medicine, University of Southampton, Southampton, SO16 6YD, UK

**Keywords:** epigenome-wide association study (EWAS), developmental trajectories, DNA methylation, DNA methylation association studies (methWAS), epigenetic epidemiology, gene × environment (GxE)

## Abstract

Genome-wide association studies had a troublesome adolescence, while researchers increased statistical power, in part by increasing subject numbers. Interrogating the interaction of genetic and environmental influences raised new challenges of statistical power, which were not easily bested by the addition of subjects. Screening the DNA methylome offers an attractive alternative as methylation can be thought of as a proxy for the combined influences of genetics and environment. There are statistical challenges unique to DNA methylome data and also multiple features, which can be exploited to increase power. We anticipate the development of DNA methylome association study designs and new analytical methods, together with integration of data from other molecular species and other studies, which will boost statistical power and tackle causality. In this way, the molecular trajectories that underlie disease development will be uncovered.

**Figure 1.**
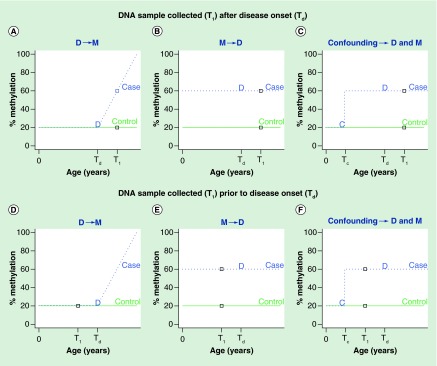
**Simplified DNA methylation trajectories for a subject without (green solid line) and with disease (dotted blue line) where (A & D) methylation changes as a consequence of disease, or (B & E) disease occurs as a consequence of methylation, or (C & F) there is no causal relationship between disease and methylation (a confounding factor independently affects both disease status and methylation), respectively.** T1 and Td represent times when DNA sample is collected and disease occurred, respectively (these events are also represented with a square and ‘D’). Tc is the time when a confounding factor (e.g., environmental exposures) affects methylation (denoted with a ‘C’). In the top panel **(A–C)**, DNA sample is collected after disease onset, we observe a positive association between methylation and disease, but cannot distinguish between each of the three scenarios D->M, M-> D and confounding. In the bottom panel **(D–F)**, DNA sample was obtained prior to disease onset, allowing us to rule out D -> M, but both M -> D and confounding are still possibilities.

The influences of nature and nurture on human health have been studied for hundreds of years. In the last few decades, derivations of genome-wide association studies (GWAS) have interrogated the interplay of the two, either by incorporating genetic and environmental measures in gene × environment screens or by screening the DNA methylome (as the latter can be thought of as the product of genetic and environmental influences). Both approaches are afflicted by problems of low statistical power to detect significant associations, and in both cases increasing subject numbers to boost power has practical impediments. As we enter the era of the methylome-wide association-study (methWAS), we discuss ways to increase statistical power appropriate to these types of studies.

## Health is a result of the interaction of genes with environment

Increasingly, we understand that genetics and prior environmental exposures determine an individual's sensitivity and resistance to extrinsic influences. This can be expressed in terms of negative symptomology, for example, a genotypic group could be more sensitive to the consequences of a high-fat diet while another genotype group is relatively resistant. For example, Asians experience higher risk of hypertension, cardiovascular disease and diabetes at lower BMIs compared with other racial groups [1–3]. It can also be expressed in terms of plasticity wherein a genotypic group does worse in a ‘bad’ environment but better in a ‘good environment’. For instance, the long allele (5-HTTLPR L) genotype of the variable tandem repeat (VNTR) within *SLC6A4* is associated with an increased risk for affective disorders under adverse conditions but also with a decreased risk under more favorable settings [4]. In another example, polymorphisms in *ESR1* moderate the effects of family cohesion on age of menarche. Specifically, girls with a GG genotype at *ESR1* polymorphism rs9304799 experience puberty later in a high quality family environment and much earlier puberty in a low quality family environment. In contrast, the impact of family environment on age of menarche in AG girls is less and has no effect on age of menarche in girls with a AA genotype [5,6].

Diseases such as obesity, diabetes, hypertension, depression, schizophrenia and coronary heart disease are major public health issues with high economic costs and significant consequences on quality of life. Their pathogenesis starts long before the symptoms are apparent and their etiology comprises both genetic and environmental components. They are the consequence of the interplay of genes and environment and cannot be sufficiently explained by their separate (or marginal) effects. Detecting which environmental exposures and which genetic variants are causative and in which combinations, is an important task to enable intervention and prevention [7].

## Screens for genetic, environmental & GxE influences are afflicted by low statistical power

Screens for environmental influences on health have tended to be hypothesis driven, but hypothesis-free, large-scale screens for genetic variants have had their own productive era in terms of GWAS [8]. The number of tests inherent in a GWAS has highlighted problems of statistical power to detect significant associations. Typically millions of genetic variants are assayed exceeding the number of subjects contained within the study, leading to low statistical power. Once a statistical modeling approach is chosen, factors influencing statistical power include:

Required significance level (chosen to protect overall Type 1 error) – less stringent level implies higher statistical power.
Magnitude of true effect size – higher effect size implies higher statistical power.
Sample size – higher sample size implies higher statistical power.
Noise (unwanted variability) – more noise implies less statistical power.

A type 1 error rate of 0.05 is typically used and multiple testing corrections are applied to ensure this error rate is maintained across all tests conducted. For a GWAS, which assumes all common genetic variation has been covered, the uncorrected p-value required to claim significance for an individual test is p < 5 × 10^-8^ (this corresponds to a genome-wide 0.05 type 1 error rate maintained across one million independent tests) [9–11]. GWAS researchers have tackled the problem of a stringent required significance level, by increasing the number of subjects, either in individual studies or by combining studies through meta-analysis, thereby boosting statistical power.

However when interaction with environmental components is included in a genome-wide screen, increasing subject numbers is not always feasible. Adding more subjects for genetic characterization has economies of scale but environmental observations on more subjects do not [12]. Genetic information can be obtained from a one-time collection of DNA sample and running genotyping assays for the additional subjects incurs only incremental cost and little additional time. In contrast, collecting information on environmental exposures from additional subjects, such as measuring their fasting blood glucose levels, costs the same, has the same subject burden and takes the same amount of clinic time for subject 2000 as it did for subject 1. Also and importantly, testing for an interaction itself requires larger sample sizes to achieve the same statistical power due to additional variability associated with the estimated interaction term [13]. An oft-quoted ‘rule of thumb’ is that detection of an interaction requires a sample size at least four-times larger than that required for the detection of a main effect of comparable magnitude [14]. This may be one of the reasons why the wealth of literature on GxE effects (especially in the psychological sciences) has not translated to a raft of searches for GxE associations in genome-wide data. These studies, variously called genome–environment-wide interaction (GEWI) and gene × environment wide association (GxEWA) studies have been suggested [15,16]. However in actuality, candidate gene approaches, for example [17]; or dimension reduction using polygenic risk scores, for example [18]; or GxE test of candidates identified in a GWAS, are usually applied.

## Utilizing DNA methylation marks as a proxy for GxE

Happily, there are intermediate markers, which integrate gene and environment effects and can be tested against phenotype. Genome-wide DNA methylation marks can be assayed by microarray [19,20] (although these covers only a small fraction of the methylome) or emerging NGS approaches [21,22] and therefore methWAS can be conducted. DNA methylation marks are a product of the interaction between genes and environments. One unusually complete example is the methylation state of *FKBP5*, which is decreased in response to childhood trauma only in carriers of a risk allele. Methylation state goes on to predict stress reactivity and risk of psychopathology in adulthood [23,24]. Teh *et al*. [25] showed that the majority of variation in the neonate DNA methylome is best explained by an interaction of an SNP and a prenatal environment (compared with the main effect of a SNP or an environment).

A phenotype may be the result of many polymorphisms and environmental factors. It is difficult to model all these possibilities in a screen. However DNA methylation marks are downstream of all these factors and therefore combine multiple inputs. MethWAS can be conducted as an alternative to GEWIS, without the need to collect complex environmental data or model the different ways interactions can occur [26]. This is notwithstanding the fact that DNA methylation marks work in concert with each other and may interact to cause phenotypes. Similarly, epigenetic marks may act in concert with genetic or environmental factors to affect phenotype as elegantly described by Ladd-Acsta and Fallin [12]. There is certainly an argument for conducting methylation × genetics or methylation × environments or methylation × methylation studies. However testing methylation marks against phenotype is already a more comprehensive study of genetic and environmental influences than can be hoped for using a finite number of environmental measures and simplistic statistical models to integrate them with genotype, as would be done in GEWIS.

As has been ably covered elsewhere [27–29], researchers conducting methWAS must tackle decisions such as: the platform to use to measure the DNA methylome, the appropriate tissue to sample and the timepoint to measure both the methylome and environmental exposure or phenotype. Besides these crucial decisions to ensure a well-designed study, they must find ways of boosting statistical power and appropriately analyzing DNA methylation which is a continuous data-type (a marked contrast from categorical DNA polymorphism data). There is reason to hope that effect sizes will be larger for epigenetic marks than for genotypic ones, as some published studies seem to suggest [30–32]. Already significant and replicated associations have been reported from large studies, for example, *HIF3A* methylation associated with BMI [33] and *AHRR* methylation for smoking [34], and extended by independent groups, for example [35–39] and [40], respectively. When possible, the study should be sufficiently powered with adequate sample size. However, due to the increased difficulty and cost in sampling the relevant tissue (at sufficient depth and resolution) for methWAS compared with GWAS, it is unlikely that methWAS studies will achieve the same sample numbers as GWAS. Therefore, statistical power must be boosted by other means, by exploiting the intrinsic characteristics of DNA methylation data to reduce unwanted variability and by efficient statistical modeling.

## Increasing power of methWAS by reducing (unwanted) variability

One of the factors that negatively affects statistical power is unwanted variability. For methylation data, one of the key sources of unwanted variability is cellular heterogeneity [41–43]. The most efficient way to reduce unwanted variation caused by cellular heterogeneity is to investigate methylation in more homogeneous samples. For example, blood can be fractionated and DNA methylation investigated in specific cell types, for example [44], and more precisely from specific subpopulations, for example [45–47]. Another possibility for blood-based study is to directly measure the cell count in the DNA sample and adjust methylation data accordingly, for example [42,48]. However, such measures are not always feasible, for instance when studying previously frozen bloods or tissues that are by nature heterogeneous and not easily fractionated (e.g., placenta). In these scenarios, cellular heterogeneity has to be accounted for using statistical approaches, either by deriving an estimate of cellular proportions using an independent dataset of methylation profiles of the individual cell types and adjusting for these derived cell type proportions (reference-based adjustment) [49], or directly adjusting without inferring the cellular proportions (reference-free adjustment) [50]. These approaches, although absolutely necessary in methylome data from mixed cell type samples [42,43], have limitations. Reference-based adjustments require the use of an appropriate cell-specific methylation reference panel. When it is unclear if the available reference-panel is appropriate for the specific study (e.g., an adult blood reference panel might not be appropriate for use in investigating infant cord blood methylation [51]) it might be useful to construct reference-panels that are appropriate to the study. For example, a study investigating infant cord blood methylation might fractionate and assess methylation in a few fresh (non-study) cord blood samples to construct a reference panel, which is less tedious than fractionating or obtaining cell counts in all the study samples. It is also important to optimize the performance of this procedure by selecting the most informative cell type markers from the methylome [52]. The reference-free approaches calculated from the dataset under study, are limited in their precision and may remove (wanted) biological variability from the data and hence reduce statistical power.

Other major sources of unwanted variability in methylome data are batch effects. In methWAS known sources of batch effects are bisulfite conversion batch, experimental batch, chip and position on chip for array studies [53] and reagent set and order for sequencing runs [54,55]. Minimizing batch effects from these sources can be done by designing the study to ensure the phenotype of interest is not confounded by predictable batch effects, optimizing laboratory procedures and by statistical approaches to correct observed batch effects in the data [20]. There is scope to further improve the processing of DNA methylation data. For instance current processing methodologies for Illumina450K data assume that the methylated and unmethylated signals form independent gamma distributions, which is obviously not true [63]. Last, there are known sources of variability that are not necessarily the variable of interest, such as sex [56], ethnicity [42] and age [57,58], which can be adjusted for in downstream analyses.

## Increasing power of methWAS by appropriate statistical modeling

At an individual site, in an individual cell, methylation is either 0 or 100%. However in tissue samples, a mixture of cells is assayed to give an average methylation percentage at any given site. Thus percent methylation values are continuous and range from 0 to 100. Methylation levels at particular sites are often not normally (Gaussian) distributed across samples; they can be bimodal, profoundly skewed, or multimodal. Extreme values of highly methylated and highly unmethylated sites show reduced variance compared with intermediate values [59,60]. Both of these facts violate assumptions made by classical statistical techniques such as ordinary least squares regression which assume normality and constant variance of model residuals (errors) [61]. Violation of statistical assumptions can lead to large numbers of false-negative results and therefore loss of power [62]. Various approaches can be used to surmount problems arising from methylation value distributions. One solution is to logit transform the percent methylation values (often termed beta values) to M values [59] but this does not always result in an appropriate error distribution with constant variance [63]. Other solutions include modeling using robust regression to minimize the effect of outliers or robust standard errors for regression coefficients to deal with heteroskedasticity [62], modeling using non-normal errors [63,64] or using nonparametric techniques which rely on ranks rather than the methylation values [65]. Bayesian approaches have also been used to shrink estimated sample variances toward a pooled estimate [66].

Many modern platforms for methylome assay (e.g., methyl-capture sequencing, Infinium arrays) measure methylation sites at single base resolution. Most published methWAS analyzed each site individually for association with exposure/phenotype of interest, for example [22,33]. The individual site analysis is then corrected for multiple testing using Bonferroni [67] or false discovery rate (FDR) [68,69]. Recently, a new approach to FDR that is less stringent has been suggested [70]. Its appropriateness in methWAS hinges on the assumption of unimodality, in other words, whether the effect size has a common mode. The assumption might be violated when comparing old age to young age samples because there are global methylome changes with aging, where both hypomethylation and hypermethylation can occur. However, it may be reasonable for phenotypes, which have subtle locus-specific effects on the methylome.

As an alternative to individual site analysis, grouped-site analysis, where one assesses the collective association between a group of sites and phenotype, can be employed. The advantages a grouped analysis confers over individual marker analysis have been well investigated in the context of GWAS and many of the same reasons apply here. Briefly, an individual marker analysis could be an erroneous signal or the analysis suffers from power loss because the testing procedure ignores the correlation of the tested markers and does not allow joint effects of marks to be modeled. The inefficiency due to multiple testing becomes particularly relevant as the number of sites (in methWAS usually CpGs) being tested increase with the newer arrays and sequencing platforms. For example, the widely used Illumina Infinium HumanMethylation450 beadchip array includes 485,512 sites [19] while the new Illumina Infinium MethylationEPIC beadchip includes 856,187 sites [71]. Sequencing technologies can in principle assay all approximately 28 million CpGs in the human genome, but in practice are likely to assay an order of magnitude less (a recent study using methyl-capture sequencing of clinical samples assayed approximately 2–3 million methylation sites [72]). As methylation at CpGs show patterns of correlation across the epigenome, in principle, we can estimate the effective number of independent tests/CpGs across the epigenome and adjust for this number, as opposed to all the 2–28 million CpGs, similar to what was done in GWAS [9–11]. However, this is not a trivial task as, unlike genotype, DNA methylation is variable across cell-type, tissue and age so this estimate would have to be performed for each combination of cell combination, tissue and age tested.

Grouped-CpG analysis decreases the number of tests by aggregating co-varying CpGs. This is especially attractive, for higher coverage methods to reduce the number of tests while retaining the broader survey of interindividual variation. Another advantage is that multiple-testing corrections often assume independence between tests and grouping-CpGs improves the fit of the data to this assumption. Existing grouped-CpG analysis can be classified into two classes based on how the groups are defined: groups are defined in testing procedure and groups are formed *a priori*. Methods that group the CpGs during the testing procedure include region discovery [73], bump hunting [74] and comb-p [75]. These tests typically first test each CpG for association between exposure/phenotype, and then define the region of differential methylation using the individual marker analyses. They have the advantage of prioritizing association with the phenotype to form the groups. However, when the groups are defined as part of the testing procedure, computationally intensive permutation procedures are generally required to obtain a p-value that maintains the correct Type 1 error control [76]. On the other hand, when the groups are defined and formed *a priori* (i.e., without using exposure/phenotype to define the groups), multiple testing correction is straightforward via standard procedure over the total number of groups tested, providing a computationally efficient alternative. Examples of tests include forming groups using genomic annotations [77], such as genes, pathways or CpG islands. For example, if groups were formed using CpG island context (˜29K CpG islands in genome, each island consisting of all CpGs within that island would represent one test), the bonferroni threshold to maintain a Type 1 error rate at 0.05 would be 0.05/(29 × 10^3^). However, this approach risks reducing power, unless the collapsing approach is carefully chosen (see next paragraph). Alternatively, groups can be formed using the methylation data to find spatially correlated CpGs data such as in adjacent sites clustering (A-clustering) [78], or using methylation data to group CpGs based on co-varying networks as was done in weighted correlation network analysis (WGCNA) [79]. The choice of how groups are formed can affect both the statistical power and interpretation of results. The ideal choice remains an open research question, and is likely to depend on the choice of platform for methylation assay which determines the scarcity of the CpG measures as well as underlying CpG co-varying architecture and phenotype under study. For example, combining a CpG with an effect on phenotype with other non co-varying CpGs that do not affect the phenotype could introduce substantial noise and reduce the power.

Another factor that affects statistical power, is how information is aggregated across the CpGs within the group. In the simplest case, information is collapsed at the CpG level into a single summary variable (e.g., mean methylation) [80,81]; and one tests for association between this summary variable and the phenotype. This test highly resembles the collapsing/burden tests used in the analysis of rare variants sequencing association studies (an example of a collapsing test is to use the mean or total number of rare variants in the group as a summary variable), and implicitly assumes that all CpGs within the group share the same effect size (and hence can be represented using a single regression coefficient) [82,83]. However, if the effects of the CpGs on phenotype have opposite signs or if only few CpGs within the group show association with the phenotype, this simple collapsing approach would be highly inefficient. Another approach to aggregating information at the CpG-level is to conduct a principal component analysis (PCA) on the CpGs within the group and test the resulting principal components (PC) for association with the phenotype. WGCNA [79] utilizes such an approach where the first PC for CpGs within the module (denoted the eigengene/eigenCpG) is compared with the phenotype. This analysis offers the chance to determine ‘emergent properties’ of the data. However, the power of this approach is highly dependent on the appropriate number of PCs used, for example, if only the first PC is used and important information is captured in the second PC, this approach can suffer from power loss [84]. An alternative method is to aggregate information at the test statistics level, such as in kernel machine regression [77]. This method offers an advantage by allowing effects to have different signs and boosts power by varying the degrees of freedom of the test depending on the correlation of tested terms. Another closely related method aggregates information at the p-value level and uses weighted inverse χ^2^ method to account for correlation in the p-values [85].

## Increasing power of methWAS by leveraging data from multiple molecular species

A complementary approach to increase power in methWAS is to integrate omics data from other molecular species such as genetics, sequence metrics and chromatin states and transcriptomics since DNA methylation is influenced by genotype [25], the broader characteristic of the sequence context [86], and chromatin state [87] and both influences and is influenced by transcription [88–90]. When integrating information from the transcriptome, it is essential that the transcriptome is measured in the same tissue as interrogated for the DNA methylome and appropriate for the phenotype in question. In the simplest case, data from other molecular species is used as a filtering criterion to reduce the number of CpGs tested. For example, if genotype and methylation data are both available, analysis could be restricted to only methylation marks showing association with genotype. In this way power is increased as the number of tests is reduced. For example, in a study investigating methylation as an intermediary for genetic risk in rheumatoid arthritis, Liu *et al*. [91] required candidate CpG ‘mediators’ to have methylation levels which co-vary with in *cis* genotype. If transcription and methylation data are available, one could restrict the CpGs tested to those affecting transcription (i.e., CpG showing association between transcription and methylation) [92]. However, this approach is very conservative as a methylation mark could affect transcription in different tissues or conditions to the one tested, and could do so in a nonlinear way, which may not be detected by the analysis. So false positives are reduced at the expense of increasing false negatives. Both of these examples do not examine the association between phenotype and other molecular species. Alternatively, the associative screen is first conducted for the methylome and other molecular species separately and then the nominally significant marks are mapped to a common identifier, often genes or pathways. Identifiers that show statistical significance for multiple species are meta-analyzed and prioritized. These methods increase power by decreasing the required significance level for any one type of molecular species and instead requiring association of phenotype to be present across molecular species, for example [93,94] but again they risk missing true positive because of the stringent criteria that the molecular levels be linearly correlated in the cell type(s) and conditions tested.

The different molecular species can also be jointly analyzed in the association analysis. When jointly analyzing different molecular species with measurements of the same nature (e.g., continuous measurements for both methylation and transcription), aggregating information at the data level is possible. Methodologies suggested for this type of analysis include factor analysis performed on data from all molecular types, and then constrained (for example by linear discriminate analysis or SVA) to the phenotype [95]. Another possibility is to apply WGCNA [79] with the co-varying network modules constructed using all molecular species together (each molecular species is appropriate scaled). When jointly analyzing molecular species with different types of measurements (e.g., categorical data from genotype vs. continuous data from methylation), analysis methods that aggregate information at the test statistics-level are generally more appropriate. For example, Zhao *et al*. [77] employed kernel machine regression and constructed a test of the joint effects of a group of SNPs and a group of CpGs on a phenotype. New approaches may also integrate stand-specific information from DNA methylome and transcriptome data and detect the association of allele-specific methylation (ASM) and expression (ASE) with disease. Regions of ASM and ASE are variable across individuals (as they are often a consequence of in *cis* polymorphisms affecting DNA methylation states) and across tissues within the same individual [96] suggesting tissue-specific functional roles. Again there is an assumption that all molecular species in a network will show coordinated behavior in a manner that is amenable to the statistical tools and in the tissue and condition tested.

## Increasing power & addressing causality in methWAS by leveraging information from multiple timepoints

Simplistically there are three possibilities that can explain a true observed association between two variables (besides chance and selection bias). (i) The first variable causes the second variable; (ii) the second variable causes the first variable; (iii) a third factor (confounder) is a common cause of both variables. In interpreting findings from observational studies, one has to address the question of whether the associations are likely to be causal (scenario i or ii) or due to confounding (scenario iii). In this respect, GWAS findings are more readily interpreted because genetic variation occurs upstream of other influences and can not be influenced by them. Therefore, while causal mediation methods have been applied in GWAS [97] their application in methWAS is less straightforward. As DNA methylation is dynamic across the human lifecourse, methWAS findings have to contend with issues of confounding by genetic and/or environmental factors and, in the absence of confounding, directionality of causation. Furthermore, the causal possibilities can co-occur, for instance a methylation mark can cause disease but development of the disease can then affect methylation at the same CpG. Therefore, attempting to determine directionality of effects from a observational methWAS study with data collected from a single time point is extremely challenging. For example, Figure 1 A–C presents a simple scenario where methylation at a disease-associated CpG is stable with age and measured at/after disease onset (as happens in cross-sectional or case–control studies, for example [91]). We can observe a positive correlation between methylation and disease at T_1_ but it is not possible to discern between causality in either direction (Figure 1A vs 1B) or the confounding case (Figure 1C). If the DNA sample was collected prior to disease onset, for example in prospective cohort studies, the temporal relationship is easier to establish (Figure 1D–F) and it is possible to discern between the casual scenarios (Figure 1D & 1E). Nevertheless, it remains impossible to rule out confounding (Figure 1F). Some, studies have used Mendelian randomization [39,98] and mediation analysis [91] to address causality. These methods require strong assumptions including the assumption that the genetic instrument (or any polymorphisms they are in linkage disequilibrium with) can affect the phenotype only through methylation and not through any other pathways. They also require very large sample sizes or very high effect sizes to achieve adequate statistical power [99].

Jointly interpreting findings from multiple observational studies with different study designs could provide better clues as to the directionality of causation. In a recent study, DNA methylation was implicated as a mediator of the effects of genetic variants on the development of hypertension. SNPs significantly associated with hypertension were identified in a replicated GWAS study in adults. In a subpopulation of those adults, methylation marks in *cis* with the associated SNPs were also strongly associated with the phenotype. Additionally, in a (separate) population of neonates who are not affected by hypertension, the same methylation marks still associated with the identified SNPs. This suggests that the variation in methylation is upstream of the phenotype, not a consequence of it [100]. Data from different age groups could thus allow us to better understand the directionality of the associations. In another example, a replicated methWAS, identified the association of methylation of *HIF3A* and adult BMI [33]. The authors speculated that *HIF3A* hypermethylation was a consequence of increasing adiposity. However, the association of *HIF3A* methylation and weight was also detected in neonates suggesting it was not solely a consequence of adult acquired adiposity.

Alternatives to interpreting findings from multiple observational studies are longitudinal studies that prospectively sample the methylome and collect environmental influences at multiple time points. Longitudinal studies better allow us to map methylation trajectories alongside disease progression, and environmental influences. Therefore, they are powerful in examining causality [27,101]. This aim is central to efforts of longitudinal cohorts with epigenetic sampling [39,102–104]. Additionally, multigenerational studies will allow investigation of epigenetic mediation of transgenerational transmission, for example, of metabolic profile [105–107], lifespan [108] or psychophysiological trauma [109].

## Future perspective

Boosting statistical power in individual studies is desirable. Statistical power can be boosted by increasing sample size to logistical limits, although it is difficult to estimate the required sample size *a priori* (as is often required by grant awarding bodies) as variance of continuous methylation levels and effect size on phenotypes are usually unknown. Statistical power can be boosted through careful study design and data analysis as described here. Lessons can be learnt from the evolution of GWAS studies but DNA methylation is not analogous to DNA polymorphisms and different approaches are necessary. Statistically significant results remain vulnerable to confounding by unknown factors in the data, which could drive a spurious association between phenotype and DNA methylation marks. Replication in independent datasets, as has become standard for GWAS is perhaps even more critical for methWAS. Additionally, meta-analysis can be conducted across studies to boost the total sample size. Large-scale epigenome mapping initiatives have provided hugely important information about DNA methylation across tissues and individuals and their relationship with other molecular marks [110–112]. These provide methWAS studies with important context, which could be utilized to enhance statistical power, for instance in more appropriate grouping of CpGs and modeling of the interaction with other molecular species. As the field goes on to produce more data from samples on the continuum of health and disease, development of analytical methods for the interpretation and integration of methWAS data while conserving power, will become an even more important research challenge. MethWAS offers hope of the confident identification of DNA methylation marks, which are downstream of genetic and environmental influences but upstream of disease. Their discovery, in part relies on increasing the statistical power of methWAS.

**Executive summary**

**Health is a result of the interaction of genes with environment**

Complex diseases are a product of the interaction of genetic predisposition and environmental influences.

**Screens for GxE influences are afflicted by low statistical power**

Hypothesis-free large-scale screens for genetic variants influencing disease (genome-wide association studies [GWAS]) require thousands of subjects to achieve statistical power to detect true associations.
Investigating interactions with environmental influences in these screens, reduced power further.
Moreover, increasing number of subjects is nontrivial as collection of environmental measures (especially longitudinal measurements) is costly and burdensome.

**Utilizing DNA methylation marks as a proxy for GxE**

An alternative is to study the DNA methylome.
DNA methylation marks are putatively downstream of multiple causative genetic and environmental factors and upstream of disease.
However, as the DNA methylome must be measured in an appropriate tissue and at appropriate time(s), methylome-wide association studies (methWAS) are challenging to perform in massive numbers of subjects. Therefore, statistical power must be boosted by other means.

**Increasing power of methWAS by reducing (unwanted) variability**

A key source of unwanted variability in methWAS studies is introduced by cellular heterogeneity. This can be tackled by study design, collection of cell count data and statistical adjustment.
Other sources of unwanted variability can also be partially bested by statistical modeling.

**Increasing power of methWAS by appropriate statistical modeling**

Statistical models employed within methWAS must account for the non-normal distribution and heteroskedascity of the data.
Appropriate multiple testing procedures must also be applied, without causing excessive loss of power.
Grouping CpGs and testing grouped-CpGs as a single unit reduces the number of variables to test and so increases power.

**Increasing power of methWAS by leveraging data from multiple molecular species**

Combining methWAS with data from other molecular species (e.g. genotype and transcriptome) can decrease required significance levels and so increase statistical power.
It can also detect the ‘emergent properties’ of the data.

**Increasing power & addressing causality in methWAS by leveraging information from multiple timepoints**

Causality and confounding are important issues in methWAS.
It is challenging to address them in single timepoint data.
However, there are promising clues emerging from studies that combine observations made at different stages of the lifecourse.
We look forward to longitudinal and multi-generational studies that incorporate DNA methylation measures.

**Future perspective**

Replication across independent studies and sample sets is critical for the future of methWAS, especially to address confounding.
We look forward to taking full advantage of existing datasets to boost the power of methWAS studies and uncover molecular trajectories that underlie disease.
